# Relationship of National and State Quarantine

**Published:** 1895-04

**Authors:** 


RELATIONSHIP OF NATIONAL AND STATE QUARANTINE.—It will be remembered by readers of the TEXAS MEDICAL JOURNAL that in our February issue there was an editorial under the head of "A Specious Plea," in which we took issue with our neighbor, the *Texas Sanitarian*, on the above subject,—"The proper relationship of State and National Quarantine." The *Texas Sanitarian* advocated a complete surrender of control of all the State quarantines, unasked, to the national government; i. e., to the Marine Hospital Service. The basis for this proposition was the statement of Dr. Jerome Cochran, of the Alabama State Board of Health, in the *Alabama Medical Age* of November, 1894, and quoted by the *Texas Sanitarian*, in effect that the Mobile quarantine is not subject to control of either State or local board of health, but is a "close corporation," composed of business men, who would not enforce strict quarantine measures if it interfered with commerce; in effect, that Mobile was practically without quarantine protection, and constituted, therefore, a standing menace to the other States, etc. The *Texas Sanitarian* further pleaded, as a reason for surrendering State control to the United States government, that there is no uniformity in quarantine regulations.

This position we controverted, and sought to show that the predicate upon which the argument was based was fallacious, and therefore that the deductions amounted to nothing. We denied the propositien that the Mobile quarantine is weak and inefficient to protect; and while we were not able otherwise to demonstrate it, we cited the law to show that such condition as was represented to exist at Mobile is inconsistent with the ordinary discharge of duty by the Surgeon-General of the Marine Hospital Service; that either the regulations at the Mobile quarantine are as strict as at other ports, and they are as rigidly enforced as at any other station, else the Surgeon-General is guilty of gross neglect of duty; for it is especially made his duty to see that all State and local quarantines are efficient, and if not, to make them so.

The question was submitted to the Surgeon-General of the Marine Hospital Service, and we have the pleasure of giving herewith his reply, conveyed in a courteous letter under date of March 18 (ult). The Surgeon-General says:

* * * "Replying to the questions in your letter of the 11th instant, *seriatim*, I have to state, in answer to the first question, namely: 'Is Mobile practically without quarantine protection, and are the other States thereby endangered'?—that the written report of Surgeon W. H. H. Hutton, of the Marine Hospital Service, upon the quarantine station at Mobile, in accordance with printed instructions (a copy of which I enclose for your information), contains the assertion that 'the quarantine facilities [at that port] are ample for the care of such shipping as comes to that port'; and I will further add that *other States are not endangered* by the character of this quarantine. [Italics ours.—ED.]

"As to the rules and regulations governing coast quarantine, you are informed that they are practically uniform. * * * The method of enforcing this uniformity is seen in the printed instructions to medical officers inspecting local quarantines, already referred to. * * *

"With regard to your inquiry, 'Have not State rules, where not in accord with, and up to the requirements of the Marine Hospital Service, been amended or re-inforced by others, promulgated by the Marine Hospital Service'? I would state that all State quarantines have readily agreed to carry out the regulations prepared by the Marine Hospital Service for the government of quarantines.

"Finally, it may be added, that the law provides for the *assumption* of *quarantine control by the United States wherever it may be inadequate* [as contended by us.—ED.], and in this event, an appropriation is available; BUT for the performance of quarantine functions *under other circumstances*, specific appropriations are [have to be?] made."

It will thus be seen that the TEXAS MEDICAL JOURNAL is fully sustained by the Surgeon-General in the position taken, in every particular; and that we have made good the assertion that it "aint so," with regard to our neighbor's predicate, thus destroying the force of his argument.

